# Acute versus delayed total hip arthroplasty after acetabular fracture fixation: a systematic review and meta-analysis

**DOI:** 10.1007/s00590-023-03489-y

**Published:** 2023-02-22

**Authors:** Kaifeng Liang, Muhammad Haseeb Gani, Xavier Griffin, Paul Culpan, Takura Mukabeta, Peter Bates

**Affiliations:** 1 Barts and The London School of Medicine and Dentistry, Queen Mary University of London, The Royal London Hospital, Whitechapel Road, London, E1 1FR UK; 2 Bone & Joint Health, Barts Health NHS Trust, London, UK

**Keywords:** Acetabular fracture, Pelvic fracture, Fix and replace, Total hip arthroplasty, Total hip replacement, Trauma

## Abstract

**Background:**

Post-traumatic osteoarthritis (PTOA) is a disabling complication of open reduction and internal fixation (ORIF) for acetabular fractures. There is a trend towards acute total hip arthroplasty (THA), ‘fix-and-replace’, in patients considered to have a poor prognosis and likelihood of PTOA. Controversy remains between early fix-and-replace, versus delayed THA as required after initial ORIF. This systematic review included studies comparing functional and clinical outcomes between acute versus delayed THA after displaced acetabular fractures.

**Methods:**

Comprehensive searches following the PRISMA guideline were performed on six databases for articles in English published anytime up to 29 March 2021. Two authors screened articles and discrepancies were resolved by consensus. Patient demographics, fracture classification, functional and clinical outcomes were compiled and analysed.

**Results:**

The search yielded 2770 unique studies, of which five retrospective studies were identified with a total of 255 patients. Of them, 138 (54.1%) were treated with acute and 117 (45.9%) treated with delayed THA. Delayed THA group represented a younger cohort compared to the acute group (mean age, 64.3 vs 73.3). The mean follow-up time for the acute and delayed group was 23 and 50 months, respectively. There was no difference in functional outcomes between the two study groups. Complication and mortality rates were comparable. Delayed THA had a higher revision rate compared to the acute group (17.1 vs 4.3%; *p* = 0.002).

**Conclusion:**

Fix-and-replace had functional outcomes and complication rates similar to ORIF and delayed THA, but lower revision rates. Although the quality of studies was mixed, sufficient equipoise now exists to justify randomised studies in this area.

**PROSPERO registration**: CRD42021235730

## Introduction

Open reduction and internal fixation (ORIF) is the standard of care for displaced acetabular fractures [1]. Post-traumatic osteoarthritis is the most common mode of failure, with an incidence of subsequent total hip arthroplasty (THA) at 10-years ranging from 8 to 35%, depending on the fracture type and patient age, even in expert hands [2–5]. THA after previous ORIF is well established as the salvage surgery for painful, post-traumatic osteoarthritis (PTOA) and although it does not carry quite the same functional improvement as it does for non-traumatic OA, successful outcomes are nonetheless described across multiple studies [6, 7]. Consensus now exists across multiple studies that have identified negative prognostic factors which strongly predispose patients to necessitating THA. These include older age (> 40 years); initial fracture displacement > 2 cm; marginal or dome impaction (e.g. ‘gull-sign’); femoral head damage; dislocation of the femoral head; pre-existing OA; delayed surgery (> 2-weeks); and non-anatomic reduction [8–13]. The vast majority, 88.3% according to one study of patients who go on to require THA after acetabular fixation, do so within the first 2-years [8].

For patients who present with fracture patterns known to carry a poor prognosis, acute THA with ORIF, either at the same sitting or staged by up to 3-weeks, is gaining popularity. The described benefits are early full weight-bearing and avoidance of another major operation [14–16]. Particularly, when these risk factors are seen in older patients, this so-called fix-and-replace approach is becoming common practice, despite there being limited comparative evidence to justify it. While it may seem intuitive to treat more severe acetabular fractures with acute THA, rather than the traditional ORIF followed by subsequent THA if required, the question remains unanswered as to whether there is a difference in outcome.

The aim of our systematic review was to establish how the outcomes of acute THA compared with the traditional delayed THA after acetabular fracture fixation.

## Methods

The preferred reporting items for systematic reviews and meta-analyses (PRISMA) guideline were adhered to throughout this review [17]. The study was registered on PROSPERO (CRD42021235730). A comprehensive literature search of EMBASE, MEDLINE, PubMed, Web of Science, Scopus, and Cochrane Clinical Trials was conducted for articles published anytime up to 29 March 2021. The reference lists of recent reviews on the same topic were also examined to identify additional studies. Two reviewers (KL and MG) independently assessed all included publications to prevent the exclusion of any relevant studies.

The search strategy consisted of strings [(fracture* OR injur*) AND ‘acetabul*’] AND (‘total hip arthroplast*’ OR ‘total hip arthroplasty*’), which returned 2770 unique studies. EndNote X9 (Clarivate Analytics, Philadelphia, USA) was used to screen titles and abstracts, after which 155 studies remained. These were assessed in full-text by KL and MG to identify five studies which were included in this systematic review. The selection criteria encompassed studies that described and compared acute and delayed THA as a treatment of acetabular fractures, with a minimum follow-up period of 12 months. Three weeks from injury is the usual time period within which the THA is termed acute [18, 19]. Studies that examined only acute or only delayed treatment of acetabular fractures, systematic reviews, expert opinions, and publications not in English were excluded. Due to the limited number of primary studies on this topic, no restrictions were imposed on study design.

As the studies were predicted to be non-randomised in nature, all five studies included in the review were assessed for their quality using the Quality Assessment Tool for Observational Cohort and Cross-sectional Studies by National Heart, Lung, and Blood Institute [20]. This quality assessment tool contains 14 individual questions to help evaluate the internal validity of a study (score made out of “good”, “fair”, and “poor”), therefore informing us of the risk of bias associated with each one. The greater the risk of bias, the lower the quality rating of the study. All included studies were quality assessed independently by KL and MHG.

Data were extracted from the studies and entered into a spreadsheet based on the Cochrane data extraction tool. The following data were collected from each study: patient demographics and clinical characteristics, types of acetabular fractures based on the classification by Letournel and Judet [21], surgical details including the types of implants used, operative time, and blood loss. Follow-up data are also recorded, including functional outcome, complication rate, and revision rate. Some variables were not provided in all studies, such as Harris Hip Score (HHS), Oxford Hip Score, and surgical details. Therefore, each specific analysis was performed individually and studies were excluded from an analysis if information from that data point was not provided. Statistical analysis was performed using SPSS (IBM SPSS Statistics for Macintosh, Version 27). Demographic characteristics and numerical variables were presented with descriptive statistics, while complication rates and revision rates were analysed using a random effects model of proportions when heterogeneous and a fixed effect model when not heterogeneous.

## Results

**Fig. 1:**
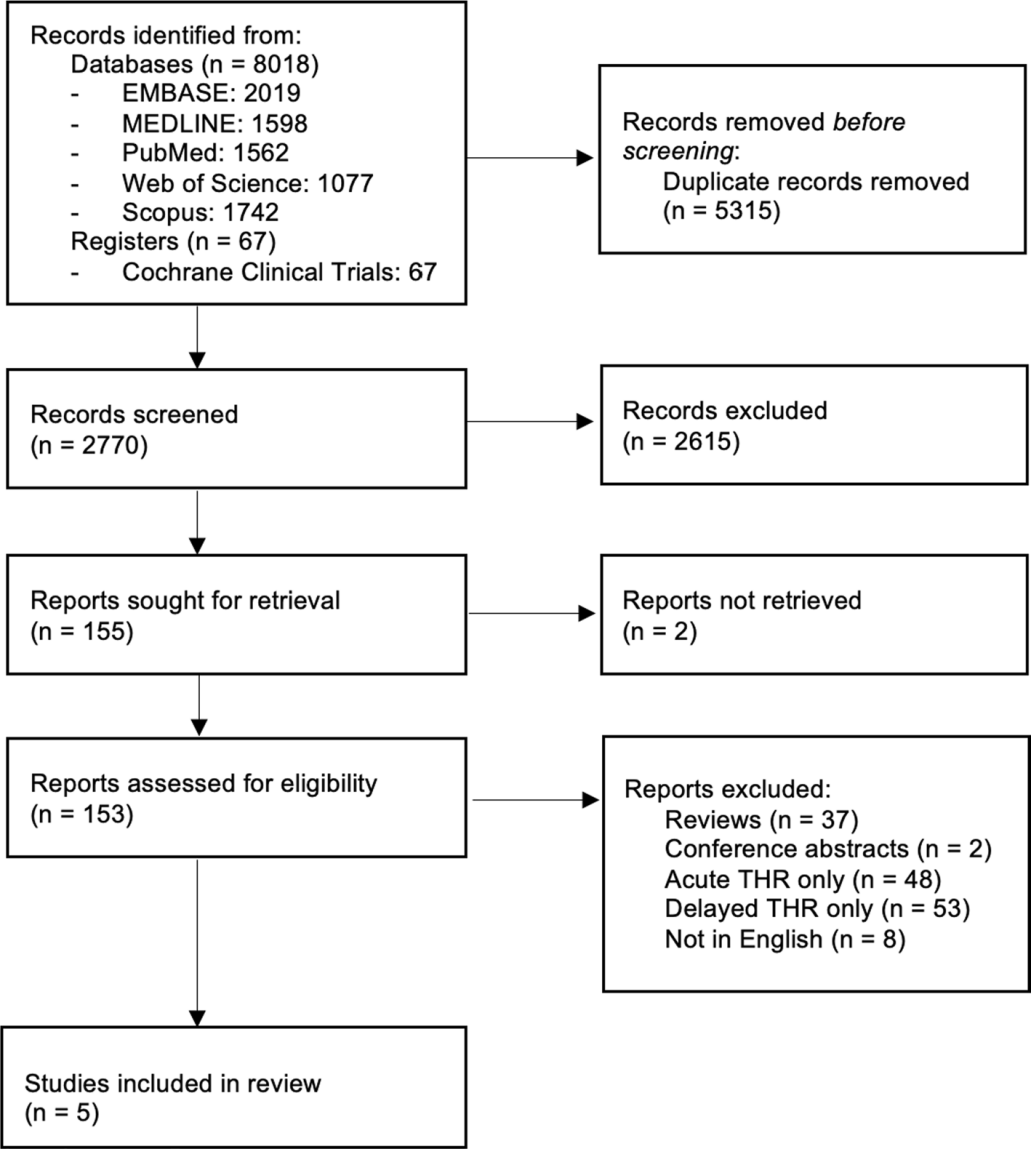
Flowchart of study selection [17]

Five studies evaluating a total of 138 acute THAs and 117 delayed THAs as a treatment for acetabular fractures were included in the final analysis (Fig. 1). The study details and demographics are shown in Table 1. The mean patient age (in years) was 73.3 (range of the means, 28–93) for the acute THA group and 64.3 (range of the means, 28–93) for the delayed THA group. The mean follow-up time (in months) was 23 for the acute (range of the means, 1–82) and 50 for the delayed THA group (range of the means, 9–113). Table 2 details the indications for performing acute and delayed THA with acetabular ORIF in the studies included.

**Table 1:** Study details and demographics

			Acute				Delayed			
	Period of data collection	Quality of study	Number (*n* = 138)	Female (*n*)	Age (years)	Follow-up (months, range)	Number (*n* = 116)	Female (*n*)	Age (years)	Follow-up (months, range)
[23]	2003—2014 (Australia)	Good	8	3 (38%)	77.6 ± 10.6	19.2^b^ (IQR 4.0, 24.7)	17	5 (31%)	71.1 ± 7.2	22.8^b^ (IQR 13.0, 39.2)
[24]	2007—2018 (Canada)	Good	12	6 (50%)	81 (68—93)*	60 ± 48ª	14	6 (43%)	76 (60—93)*	60 ± 48ª
[25]	2008—2017 (Finland)	Fair	34	10 (29%)	70 (56—87)	15 (95% CI 1—82)	9	3 (33%)	65 (58—74)	72 (95% CI 15—113)
[26]	2003—2010 (Canada)	Good	20	–	60 (28—89)*	31.2 (12—79.2)	20	–	60 (28—89)*	28.8 (9—62.2)
[27]	1983—2003 (Belgium)	Fair	64	56/121 (46%)ª	78*	30.7 (12—180)ª	57	56/121 (46%)ª	53*	30.7 (12—180)ª

*Significant difference between the acute vs delayed group (*p* < 0.05)

^b^Number presented is median (not mean)

^c^Quality of study based on quality assessment tool for observational cohort and cross-sectional studies by National Heart, Lung, and Blood Institute20, refer to Appendix 1 for full information

ªFollow-up data for both groups combined in the original publication (sex and follow-up time)

**Table 2:** Indications for acute and delayed total hip arthroplasty

Indications for acute THA	Authors
Displaced acetabular fracture with intra-articular comminution and/or protrusion	Chémaly, Lont
Acetabular impaction involving weight-bearing zone	Chémaly, Lont
Femoral head cartilage loss, impaction, or fracture	Chémaly
Pre-existing, severe hip osteoarthritis or avascular necrosis of the femoral head	Chémaly
Radiographic evidence of osteopenia/osteoporosis	Chémaly
High age with extensive osteoporosis	Sermon
Combined acetabular and femoral neck fractures	Sermon
Pathological fractures	Sermon
*Indications* *for* *delayed* *THA*	
Development of symptomatic post-traumatic/postoperative arthritis or avascular necrosis of the femoral head	Navarre, Nicol, Chémaly, Sermon
Postoperative dislocation	Navarre
Neck of femur fracture	Navarre
Loss of implant fixation	Navarre
Hardware breakage	Navarre
Kellgren-Lawrence score ≥ 3 (an osteoarthritis score, range 0–4) [22]	Nicol

Appendix 1 summarises the outcome of the risk of bias assessment with positive and negative comments where appropriate. Three of the 5 studies were given a quality rating of “good”, with low risk of bias. Two out five scored a “fair” in quality rating, mainly because the methods were not sufficiently explained to award them a low risk of bias.

**Fig. 2:**
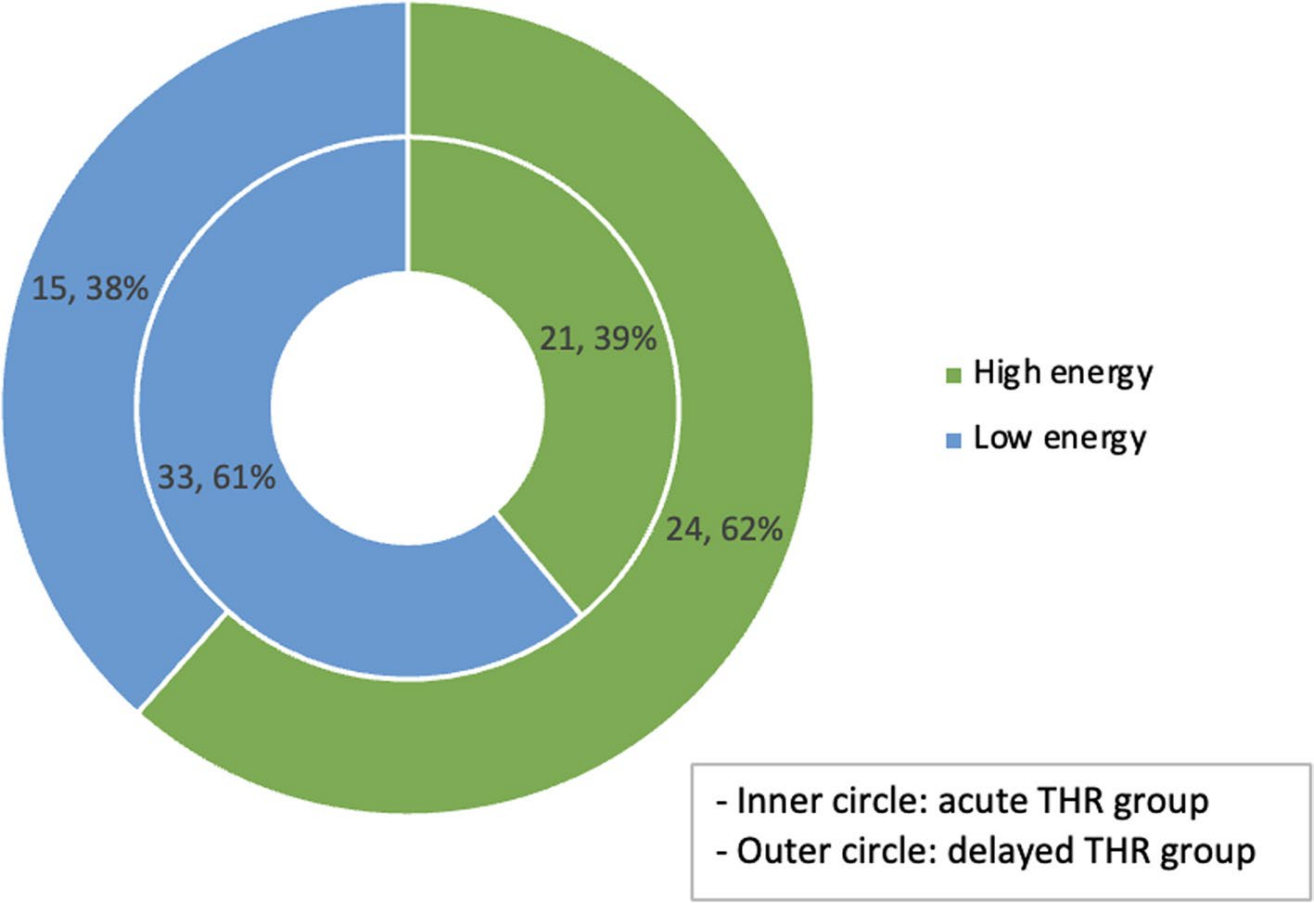
Mechanism of injury *Notes* *PW* posterior wall; *PC* posterior column; *AW* anterior wall; *AC* anterior column; *TR* transverse; *PCPW* posterior column and posterior wall; *TT* T-type; *ACPH* anterior column posterior hemitransverse; *ABC* associated both column; *TPW* transverse and posterior wall

**Fig. 3:**
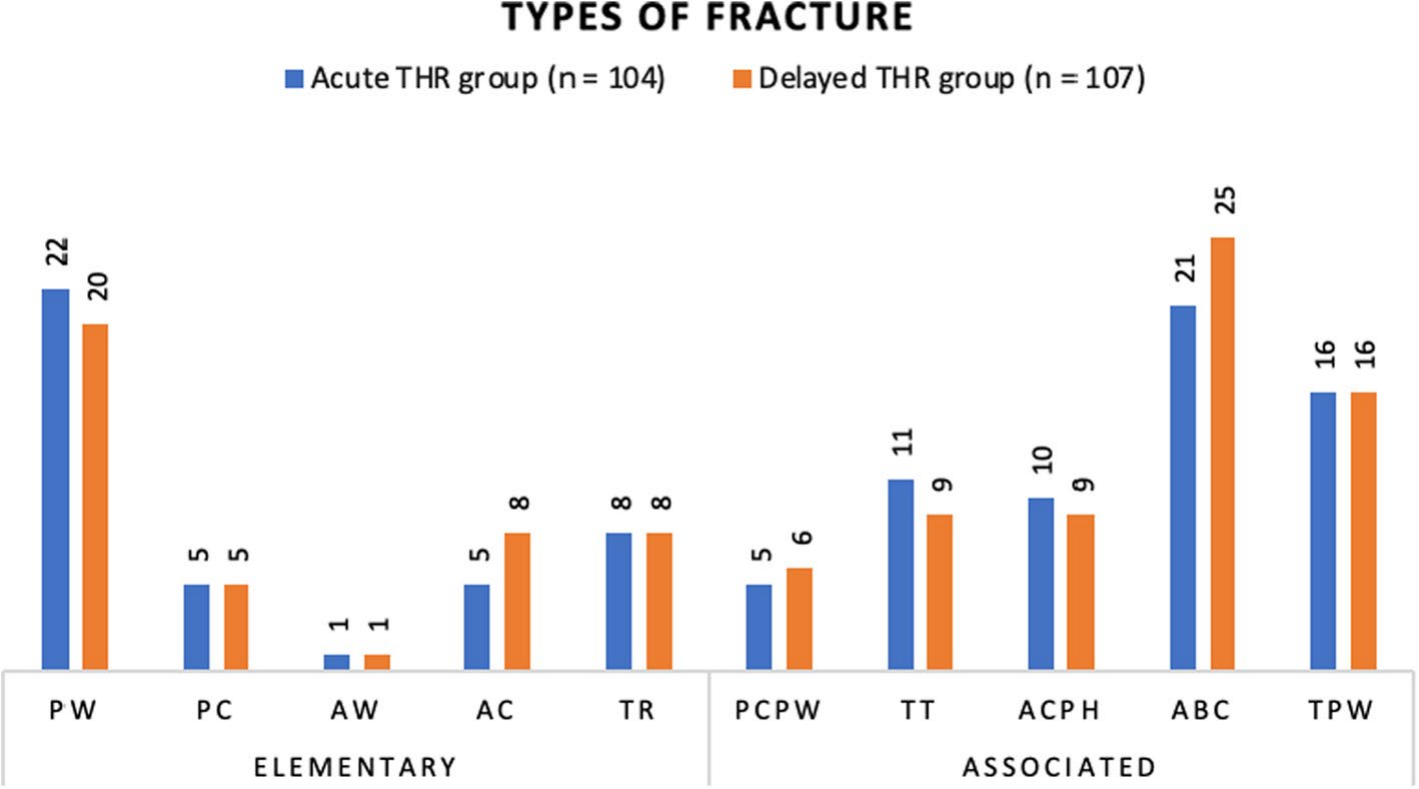
Types of fractures

Three studies provided the mechanism of injury between groups (Fig. 2) [23–25]. More acetabular fractures from high-energy injury were included in the delayed THA group (24/39, 61.5%), and more low-energy fractures were included in the acute THA group (33/54, 61.1%). All five studies recorded the type and subtype of the acetabular fracture in accordance with Letournel and Judet classification [21], however, Lont et al. did not have data for the delayed group, and therefore, their study was excluded in this analysis. In the remaining four studies (*n* = 211), 83/211 (39.3%) were elementary fractures and 128/211 (60.7%) were associated (comminuted) fractures. For both acute and delayed THA groups, posterior wall fracture was the most common type of elementary fracture and associated both column was the most common type of associated fracture (Fig. 3).

Specific implant types were described in two studies and based on that information, it appears that the acute THA group almost exclusively received revision-type implants (45/46) [24, 25]. The pattern in the implants used in the delayed group is less clear but Nicol describes using primary implants in 12 out of 14 cases. (Table 3).

**Table 3:** Implant types

	Acute THA	Delayed THA
Nicol et al.	Revision-type acetabular components in 11/12	Revision acetabular components in 2/14
Lont et al.	Revision-type acetabular components in 34/34	Various implants used but no individual breakdown (*n* = 9)

**Fig. 4:**
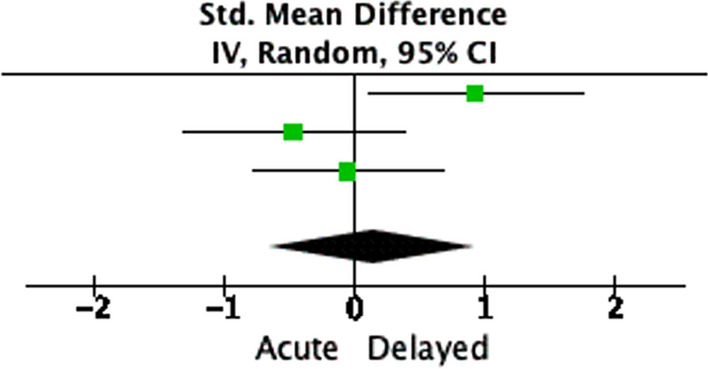
Functional outcomes

The standardised mean difference in three studies using Harris Hip Score and Oxford Hip Score was 0.14 (95% CI -0.64, 0.92; *p* = 0.73), showing no significant difference in functional outcomes between the two study groups (Fig. 4) [23–25].

Using a random effects model, complication rates were comparable between acute and delayed groups (12.2% vs 11.7%; *p* = 0.91) (Table 4). The most common complications were thromboembolic events (3/73, 4.1%) in the acute THA group, and infections (4/60, 6.7%) in the delayed THA group. Heterotopic ossification (HO) was common in both groups, slightly higher for the delayed THA group although not statistically significant (51.0% vs 59.3%, *p* = 0.22). Revision rates were higher for the delayed group (17.1 vs 4.3%; *p* = 0.002). Only two studies outlined the reasons for revision surgery. In the acute THA group, the reasons for revision were periprosthetic fracture and prosthetic joint infection, while deep infection (2 cases), non-specific infection, periprosthetic fracture, and failed cup fixation were the reasons in the delayed THA group. There was no difference in the mortality rate between the two groups (17.9% vs 10.8%, *p* = 0.60). Forest plots are shown in Appendix 2.

**Table 4:** Summary of complication, revision, and mortality rate

	Acute THA	Delayed THA	*p*
Complication rate (*n* = 134 [23–26])	9/74 (12.2%)	7/60 (11.7%)	0.91
Infection	2	4	0.59
Periprosthetic fracture	2	1	0.8
Dislocation	2	0	0.67
Thrombotic event	3	1	0.31
Loss of cup fixation	0	1	0.54
HO formation (*n* = 212 [23, 24, 26, 27])	53/104 (51.0%)	64/108 (59.3%)	0.22
Revision rate (*n* = 255 [23–27])	6/138 (4.3%)	20/117 (17.1%)	0.002**
Mortality (*n* = 65 [23–26])	5/28 (17.9%)	4/37 (10.8%)	0.60

## Discussion

Since the work of Judet and Letournel, surgical treatment of acetabular fractures has become the standard of care [21]. However, despite predominantly good long-term outcomes being reported, certain fracture characteristics have been shown to consistently predispose towards poor outcomes. In their large series of over 800 patients, Matta and Tannast devised a nomogram predicting the need for THA within two years of index ORIF based on a number of specific risk factors being present [8]. Clarke-Jenssen took this further, suggesting that if multiple risk factors were simultaneously present (patients over the age of 60; femoral head damage; and acetabular impaction) they reported a 100% conversion rate to THA at three-years [28]. Surgeons therefore feel increasingly confident in predicting which patients are liable to end up requiring THA and which will not, based upon a patient’s age, their fracture characteristics and the surgeon’s ability to obtain an initial anatomic reduction.

Two distinct patient populations are emerging in clinical practice: older patients who tend to sustain associated fractures following low-energy falls, and younger patients involved in higher energy trauma with a spectrum of fracture types (Fig. 3). With an enlarging population of older people, the former group now represents the bigger subset of patients who present with factors associated with unfavourable outcomes [18]. Patients over the age of 60 years historically comprised about 11–18% of all acetabular fractures [21, 29, 30] and more recent articles report a strong increase in incidence among elderly subjects sustaining such injuries of up to 64% [31–33]. When treated with ORIF, the proportion requiring THA for post-traumatic arthritis can range 22–64% at 3 years [2–4, 34–36].

Performing early THA as part of the initial reconstruction confers the perceived benefit of early mobilisation, particularly in older patients, while simultaneously obviating the need for further major surgery [15, 36, 37]. This has been used as the driving argument to perform acute THA in carefully selected patients, generating a large number of descriptive case series and analyses looking at outcomes of acute THA [14–16, 18, 19, 36, 38]. However, while there is a surge in clinical enthusiasm for early THA in patients who seem likely to fail conventional ORIF, it remains unclear how this affects the outcome of the resulting hip arthroplasty, since most of those case series are non-comparative.

The research question behind this systematic review asks whether THA (after acetabular fracture ORIF) carries a better clinical and functional outcome if performed acutely or delayed. Our main findings are that although functional outcomes between delayed vs acute THA are equivalent, the delayed group has a significantly higher revision rate. Inconsistency in systematic reporting outcomes in the included studies made it difficult for data extraction and interpretation, but we were able to carry out a meta-analysis with the best available data.

### Fracture types

All studies used the system of Letournel and Judet to classify the initial injury pattern and we found fracture types were similar between the groups (early vs late THA) [39]. There were more associated fracture patterns (60%) than elementary (40%) overall. The mean age of patients that underwent an acute THA was 73.3 years and that for delayed THA group was 64.3 years. Similarly, the mechanism of injury was predominantly high energy (61%) in the delayed group, vs predominantly low energy (62%) in acute group. This correlates well with age variation between the groups, as older patients are more likely to sustain displaced fractures following low-energy falls, along with less reliable articular reconstruction due to poor bone quality. The reasons for acute THA included articular comminution, poor reduction, marginal impaction of the weight-bearing dome, medial protrusion, femoral head fracture or cartilage loss, high age, osteoporosis and pre-existing arthritis. These indications are similar to those in other existing studies [10–13, 38].

### Implants used

Two of the included studies described the implants used in detail (Table 3) [24, 25]. In the study by Nicol et al., 11 of the 12 acetabular components used for the acute group were revision-type implants, whereas only two of the 14 in the delayed group were revision cups. Lont et al. used a Gap II reinforcement ring (Stryker, Mahwah, NJ, USA) in all of their acute THA cases (34/34), but they did not describe which acetabular components were used in the late THA group. This bears out in existing studies by Borg et al. and Boelch et al. who used a Burch-Schneider cage (with or without additional plate fixation) to reconstruct the acetabulum in all acute THA cases in their respective series. In contrast, Lai et al. used primary acetabular components in their series of 31 patients who underwent a delayed THA for post-traumatic arthritis following previous acetabular fracture ORIF [36, 38, 40].

It is interesting that more primary implants were used in the delayed group, and this also reflects the current authors’ anecdotal clinical practice. A plausible explanation is that when the acetabular fracture heals after ORIF, the overall anatomy and continuity of the bony columns is sufficiently restored to accept a primary acetabular component. In an acute fix-and-replace setting, however, fixation restores the anatomy but revision components are still required to optimise stability in the context of the underlying acetabular discontinuity. Acute THA is therefore likely to be more expensive and more technically complex, in terms of implants used, compared with delayed.

### Functional scores

Harris and Oxford Hip Scores were used by 3 and 2 of the analysed studies, respectively; however, only three studies contained details that could lend themselves to the meta-analysis [23–25]. Based on this, we found no difference in the functional scores between the two groups at mean follow-up (*p* = 0.73). Interestingly though, Nicol et al. reported a significantly better OHS in the acute group compared to the delayed group (*n* = 26), and they advocated acute THA in selected patients based on this [24]. Hamilton et al. defined ‘treatment success’ after THA in terms of a threshold OHS of 37.5 points [41]. This criterion has been met in all the included cohorts, except the delayed THA group in Nicol’s series. Although our analysis suggests that THA satisfaction rates after acetabular fractures are not as good as those after primary THA for non-traumatic OA, comparably successful results are still achieved with both acute and delayed THA timings [18, 42, 43].

### Complications

Of the documented complications, the overall difference between the two groups was not statistically significant in our review, being 12.2% in the acute group and 11.7% in the delayed group (*p* = 0.91). Subanalysis of complications such as infection, dislocation, implant failure and VTE yielded numbers too small for meaningful comparison. Infection rate was similar between the two groups, although previous studies have reported significantly higher rate of prosthetic joint infection (PJI) in the cohort of delayed THAs following ORIF of acetabular fracture compared to primary THA (6.9 vs 0.5%) [6]. The overall rate of HO also did not show a significant difference between the two groups in this review, all five studies being open to this analysis (51 vs 59%; *p* = 0.22). The study by Chémaly and colleagues indicated that the risk of developing severe HO was significantly increased in early (acute) THA; the other studies did not report such a difference. In an exhaustive review of HO after THA, Mavrogenis et al. found an overall incidence of 55%, which is in the same range as we found in this review [44].

### Revision

While the delayed group was younger, had lesser complex fracture patterns, tended to receive primary type acetabular components and had a slightly lower mortality rate, they had a significantly higher revision rate of 17.1% at mean follow-up, compared with 4.3% in the acute THA cohort (*p* = 0.002). Borg et al. reported zero reoperations in their cohort of 13 acute THAs (so-called combined hip procedure) at 3-year follow-up [36]. This is in contrast to Stibolt et al., whom in a meta-analysis of eight studies on delayed THA after previous acetabular fracture fixation reported a mean revision rate of 18.5% at 6-year follow-up [7].

Although delayed arthroplasty surgery might be considered a ‘primary’ THA, it is in fact akin to a revision scenario with its associated difficulties. The two main factors which contribute to it being challenging are significant distortion to the underlying anatomy due to progressive collapse of the joint and extensive heterotopic ossification. These often result in the surgical procedure being more technically difficult and prolonged, with a higher incidence of impingement due to extra bone formation (HO). These ‘complex primary’ factors may underpin the higher infection and subsequent revision rates observed in the delayed group. Regarding infection specifically, previous surgery has been shown to significantly increase the risk, which in turn translates into increased rates of revision [6]. The authors speculate that a significant number of the delayed THA group might have had unrecognised deep infection that had escaped diagnosis through standard screening tools. Any occult infection from the primary fixation surgery could hasten subsequent joint degeneration and the need for arthroplasty, ultimately resulting in failure of that procedure due to the same infection.

We found revision rates in the delayed group were significantly higher (17.1 vs 4.3%), but paradoxically, complication rates were comparable (12.2 vs 11.7%). This was likely due to the partial exclusion of one study. Sermon et al. did not categorise complications into acute and delayed subgroups but they did for revisions [27]. Therefore, complications could not be brought into the comparison (and so were excluded) but revisions were included. Their study reported “30 general complications” including dislocation, nerve lesion, and infections. If these had been added to the total number, it would have raised the overall complication rate from 16/134 (12%) up to 46/255 (18%). Overall revision rates were 26/255 (10%).

### Mortality

Two studies contained segregated mortality data, and based on these, the mortality at 5 years was 17.9% in the acute group, and 10.8% in the delayed group, although this difference was not statistically significant (*p* = 0.60) [23, 26]. Lont et al. also reported on mortality but the data for the delayed group were combined with those that had ORIF only. They reported 1- and 5-year survival of 89 and 30%, respectively, in the acute THA group and 91 and 86%, respectively, for the ORIF group which also contained the delayed THA cases. Using univariate analysis, they attributed the difference in 5-year survival to the older age and comorbid illnesses found in the acute THA group [25]. In their own study, Borg et al. reported 25% mortality at 3 years in their acute THA group, compared to 0% in the group that received ORIF alone [36].

Although not significant in our meta-analysis, the numerically higher mortality in patients following acute THA may be a reflection of this cohort being older and therefore carrying more comorbidities. Plus, their operations were carried out at the index, emergency admission with less opportunity for pre-surgical optimisation. By comparison, the delayed group was younger (64.3 vs 73.3 years) and their surgeries were done in an elective manner, presumably after medical optimisation and exclusion of those too unwell to undergo the surgery.

### Limitations

There are several limitations to this review. The five included studies had heterogeneous designs and variable follow-up and all were retrospective observational studies, thereby limiting their individual internal validity. The five studies spanned more than a decade, with one (Sermon et al.) reporting on patients from 1983 to 2003. Although the proportions of fracture types observed were consistent between studies, individual surgeon preferences and centre protocols were not consistently described and it seems likely that the surgical techniques and implants (Table 3) evolved and improved during the period covered by the studies in question. This ‘learning curve’ over time obviously affects the generalisability of our results.

We also acknowledge that the two groups we compared are not demographically the same. The acute THA group were 10-years older and more likely to have sustained low-energy falls, compared with the delayed group. This makes our comparison susceptible to unseen selection bias. Also, the primary research question of the individual studies was not uniformly comparing acute vs delayed THA. For example, Chémaly et al. were reporting primarily on the incidence of HO but their group comparison allowed inclusion to this study. This affects the reliability of the pooled data. Although functional outcomes were reported in all studies, different scales were used and there was no single outcome that unified all five studies. Furthermore, two of the studies reported some of the demographic, baseline characteristics in THA after acetabular fractures as a single cohort (e.g. sex and follow-up time), meaning that we were unable to perform subgroup meta-analysis. Two of the studies included patients treated initially non-operatively (Chémaly 4/40 and Sermon 6/121), while all others received initial ORIF.

## Conclusion

Our meta-analysis focussed on the functional outcomes, complications, and revision rates after the acute or delayed THA following acetabular fracture, all which were variably reported. The data generated suggest that both acute and delayed THA for acetabular fractures result in comparable functional outcomes and complication rates. Acute THA would intuitively be associated with higher early mortality but we were unable to demonstrate significance on this. Acute THA usually requires revision-type acetabular components, with higher cost and complexity. Delayed THA is more likely to be performed with primary implants but our meta-analysis suggests this carries a paradoxically higher re-operation rate, largely related to infection and cup loosening. We acknowledge that the robustness of these conclusions is challenged by the quality of contributing evidence. We propose that sufficient equipoise now exists to justify randomised studies on this topic so that future practice may be appropriately informed.

## Appendices

### Appendix 1 Quality assessment of studies included in the review

**Table:**

Study	Quality rating	Comments, if applicable
Navarre et al. 2020	Good	Well designed with follow-up at regular intervals (6-, 12-, 24-months) and no loss to follow-up; outcome measures clearly defined and implemented
Nicol et al. 2020	Good	Outcomes clearly defined and results well-described
Lont et al. 2019	Fair	Follow-up period not pre-specified and some inadequate follow-up (< 6 months)
Chémaly et al. 2013	Good	Confounding factors described; outcome measures clearly defined
Sermon et al. 2008	Fair	Prolonged data points (20 years) result in confounding; follow-up not described
